# Supplementary material for: Evaluating the health effect of a Social Housing programme, Minha Casa Minha Vida, using the 100 million Brazilian Cohort: a natural experiment study protocol
Source: BMJ Open. 2021 Feb 28;11(3):e041722. doi: 10.1136/bmjopen-2020-041722 (PMC8098948; doi:10.1136/bmjopen-2020-041722)
Supplement: Author's manuscript [file bmjopen-2020-041722.draft_revisions.pdf]

BMJ Open

BMJ Open is committed to open peer review. As part of this commitment we make the peer review history of every article we publish publicly available.

When an article is published we post the peer reviewers' comments and the authors' responses online. We also post the versions of the paper that were used during peer review. These are the versions that the peer review comments apply to.

The versions of the paper that follow are the versions that were submitted during the peer review process. They are not the versions of record or the final published versions. They should not be cited or distributed as the published version of this manuscript.

BMJ Open is an open access journal and the full, final, typeset and author-corrected version of record of the manuscript is available on our site with no access controls, subscription charges or pay-per-view fees (http://bmjopen.bmj.com).

If you have any questions on BMJ Open's open peer review process please email info.bmjopen@bmj.com

BMJ Open

Evaluating the health effect of a Social Housing programme, Minha Casa Minha Vida, using the 100 million Brazilian Cohort: A natural experiment study protocol

Journal:	*BMJ Open*
Manuscript ID	bmjopen-2020-041722
Article Type:	Protocol
Date Submitted by the Author:	15-Jun-2020

Complete List of Authors:	Fortes Ferrreira, Andrêa Jacqueline; Federal University of Bahia, Public Health Institute; Fiocruz Bahia, Centro de Integração de Dados e Conhecimentos para Saúde (Cidacs)<br>Pescarini, Julia; Fundação Oswaldo Cruz, Centro de Integração de Dados e Conhecimentos para Saúde (Cidacs)<br>Sanchez, Mauro; Federal University of Brasilia<br>Flores-Ortiz, Renzo; Gonçalo Muniz Institute (Fiocruz/Bahia), Center for Integration of Data and Health Knowledge (Cidacs)<br>Teixeira , Camila<br>Fiaccone, Rosemeire; Universidade Federal da Bahia<br>Ichiara, Maria Yury<br>Oliveira , Rodrigo<br>Aquino, Estela M. L.; Univ Fed Bahia<br>Smeeth, Liam; London School of Hygiene and Tropical Medicine, Epidemiology and Population Health<br>Craig , Peter ; Glasgow University<br>Ali, Sanni; London School of Hygiene & Tropical Medicine<br>Leyland, Alastair; MRC/CSO Social and Public Health Sciences Unit, University of Glasgow,<br>Barreto, Mauricio; Universidade Federal da Bahia,<br>Ribeiro , Rita de Cássia<br>Katikireddi, Srinivasa; University of Glasgow School of Life Sciences, MRC/CSO Social & Public Health Sciences Unit

Keywords:	Coronary heart disease < CARDIOLOGY, Tuberculosis < INFECTIOUS DISEASES, Tropical medicine < INFECTIOUS DISEASES, PUBLIC HEALTH, EPIDEMIOLOGY

SCHOLARONE™
Manuscripts

I, the Submitting Author has the right to grant and does grant on behalf of all authors of the Work (as defined in the below author licence), an exclusive licence and/or a non-exclusive licence for contributions from authors who are: i) UK Crown employees; ii) where BMJ has agreed a CC-BY licence shall apply, and/or iii) in accordance with the terms applicable for US Federal Government officers or employees acting as part of their official duties; on a worldwide, perpetual, irrevocable, royalty-free basis to BMJ Publishing Group Ltd ("BMJ") its licensees and where the relevant Journal is co-owned by BMJ to the co-owners of the Journal, to publish the Work in this journal and any other BMJ products and to exploit all rights, as set out in our licence.

The Submitting Author accepts and understands that any supply made under these terms is made by BMJ to the Submitting Author unless you are acting as an employee on behalf of your employer or a postgraduate student of an affiliated institution which is paying any applicable article publishing charge ("APC") for Open Access articles. Where the Submitting Author wishes to make the Work available on an Open Access basis (and intends to pay the relevant APC), the terms of reuse of such Open Access shall be governed by a Creative Commons licence – details of these licences and which Creative Commons licence will apply to this Work are set out in our licence referred to above.

Other than as permitted in any relevant BMJ Author's Self Archiving Policies, I confirm this Work has not been accepted for publication elsewhere, is not being considered for publication elsewhere and does not duplicate material already published. I confirm all authors consent to publication of this Work and authorise the granting of this licence.

For peer review only

Evaluating the health effect of a Social Housing programme, Minha Casa Minha Vida, using the 100 million Brazilian Cohort: A natural experiment study protocol

Andrêa JF Ferreira[1,2*], Julia M Pescarini[1], Mauro N Sanchez[1,3,] Renzo Flores-Ortiz[1], Camila SS Teixeira[1,2], Rosimeire L Fiaccone[1,4,] Maria YT Ichihara[1], Rodrigo Oliveira[1], Estela ML Aquino[1,2], Liam Smeeth[6,7], Peter Craig[8], Sanni Ali[6], Alastair H Leyland[8], Mauricio L Barreto[1,2], Rita C Ribeiro-Silva[1,2**], Srinivasa Vittal Katikireddi[8**].

[1] Center for Integration of Data and Health Knowledge (Cidacs), Gonçalo Muniz Institute (Fiocruz/Bahia).

[2] Institute of Public Health, Federal University of Bahia, Salvador, Brazil.

[3] Brazilian University (UnB), Brasilia, Brazil.

[4] Institute of Mathematics and Statistics, Universidade Federal da Bahia, Salvador, Brazil.

[5] Department of Economics, Federal University of Bahia, Salvador, Bahia.

[6] Department of Non-Communicable Disease Epidemiology, London School of Hygiene and Tropical Medicine (LSHTM), London, United Kingdom.

[7] Health Data Research (HDR), London, United Kingdom.

[8] MRC/CSO Social and Public Health Sciences Unit, University of Glasgow, Glasgow, United Kingdom.

***Corresponding Author:** Andrêa JF Ferreira

Email: andreaferreiracv@gmail.com

Postal address: Centro de Integração de Dados e Conhecimentos para Saúde (Cidacs), Fundação Oswaldo Cruz. Edf. Tecnocentro – R. Mundo, 121 – Trobogy, Salvador – Bahia/Brazil. Postal code: 41745-715.

****Co-senior authors**

Keywords: Cardiovascular diseases, leprosy, tuberculosis, social housing, social protection, social policies, natural experiment.

Abstract

Introduction

Social housing programmes have been shown to influence health, but their effects on cardiovascular mortality and incidence of infectious diseases, such as leprosy and tuberculosis, are unknown. We will use individual administrative data to evaluate the effect of the Brazilian housing programme *Minha Casa Minha Vida* (MCMV) on cardiovascular disease mortality and incidence of leprosy and tuberculosis.

Methods and analysis

We will link the baseline of the 100 Million Brazilian Cohort (2001-2015), which includes information on socioeconomic and demographic variables, to the MCMV (2009-2015), CVD mortality (2007-2015), leprosy (2007-2015), and tuberculosis (2007-2015) registries. We will define our exposed population as individuals who signed the contract to receive a house from MCMV, and our non-exposed group will be comparable individuals within the cohort who have not signed a contract for a house at that time. We will estimate the effect of MCMV on health outcomes using different propensity score (PS) approaches to control for observed confounders. Follow-up time of individuals will begin at the date of exposure ascertainment and will end at the time a specific outcome occurs, date of death or end of follow-up (31st December 2015). In addition, we will conduct stratified analyses by follow-up time, age group, race/ethnicity, gender and socioeconomic position.

Ethics and dissemination

The study was approved by the ethic committees from *Instituto Gonçalo Muniz* – Oswaldo Cruz Foundation and University of Glasgow Medical, Veterinary & Life Sciences College. Data analysis will be carried out using an anonymised dataset, accessed by researchers in a secure computational environment according to the Center for Integration of Data and Health Knowledge (CIDACS) procedures. Study findings will be published in high quality peer-reviewed research journals and will also be disseminated to policy makers through stakeholder events and policy briefs.

Articles Summary

Strengths and limitations of this study

1. This is the first study to evaluate the effect of a social housing programme on health outcomes at national level and is likely to be the largest of its type across the world. This would allows to assess impacts on population subgroups, including adoption of an intersectionality perspective.

2. A comprehensive assessment of health impacts is being conducted, including both infectious and Non-Communicable Diseases (NCDs).

3. Our analytical approach includes the use of Propensity Score Matching, which has the limitation of accounting for only observed variables.

4. Health and behavioural information (such as smoking status, diet and comorbidities) prior to the intervention are not available, and there is therefore a risk of residual confounding arising from inadequate comparability between exposure groups.

5. Finally, this study does not allow estimating long-term effects of MCMV on health, given the limited length of follow-up available (up to eight years).

Introduction

Housing is a basic human right and an important social determinant of health and well-being, which not only includes the guarantee of shelter, but also its quality [1,2]. Several studies have investigated the relationship between population health and housing conditions, most of them in high-income countries [1,3–5]. Residential instability, crowding, temperature, and substandard housing conditions (such as water leaks, poor ventilation, dirty carpets, and pest infestation) are associated with chronic and infectious diseases [1,2,6,7]. The neighbourhood in which a house is located has also been shown to be associated with health in studies in high-income countries [8]. Physical neighbourhood characteristics that have been associated with health outcomes include: green parks, schools, health services, sidewalks, public transport, sanitation, aesthetic characteristic and connectivity of the street, bike lanes, availability and relative cost of healthy foods and tobacco [5,8–10]. Less visible but also important are social neighbourhood characteristics, which include measures of social network and support, violence, and social capital, especially in vulnerable communities in high-income countries [3,4,11].

Taking into account these relationships, there is a policy expectation that housing interventions could contribute to improve health and reduce social inequalities, especially among the most vulnerable [1,5,12]. Despite this, we are aware of little or no robust evidence on the positive and negative effects of housing conditions on cardiovascular disease (CVD), and leprosy and tuberculosis incidence. Understanding housing impacts on health in low and middle-income countries also remains particularly poorly understood.

In Brazil, there is a double burden of infectious and chronic diseases among the poorest people and, therefore, we will evaluate the effect of Minha Casa Minha Vida (MCMV), a social housing programme, both on mortality from CVD, the leading cause of death in the country, and on infectious diseases associated with poverty, like tuberculosis and leprosy [13,14]. In Brazil, 27% of all-cause of death is attributed to CVD, with most assigned to ischaemic heart disease and stroke [15,16]. Leprosy and tuberculosis are two of the most important infectious diseases in Brazil and affect predominantly vulnerable and marginalised populations [17–19]. Brazil has the second highest leprosy incidence worldwide, with almost 30,000 cases annually [13]. Tuberculosis is also common; the country reported 72,788 new cases in 2018 and 4,534 deaths in 2017 [13].

Given the social patterning of housing access and quality in Brazilian context, housing interventions may also have important impacts on the health of disadvantaged population groups, with potential benefits for racial/ethnic minorities, women, and individuals and families of lower socioeconomic position. We therefore aim to evaluate the health effects of the Brazilian social housing program MCMV, the largest social housing program in Latin American.

Our detailed objectives are:

1. To estimate the effect of MCMV on premature cardiovascular disease, ischaemic heart disease and, cerebrovascular disease mortality;
2. To estimate the effect of MCMV on all-cause mortality;
3. To estimate the effect of MCMV on leprosy and tuberculosis incidence;
4. To investigate whether any observed effects of MCMV on cardiovascular and all-cause mortality, leprosy and tuberculosis incidence differ by population subgroups (gender, race/ethnicity, age, socioeconomic position and length of follow-up).

Methods and analysis

Study design and population

This is a dynamic, retrospective, and open cohort study that will be drawn from individuals registered in the baseline of The 100 Million Brazilian Cohort [20,21], a cohort of individuals applying for government social programmes in Brazil.

Patient and public involvement

This research was done without patient involvement. Patients were not invited to comment on the study design and were not consulted to develop patient relevant outcomes or interpret the results, since we use an administrative and deidentified dataset.

Intervention

We report intervention characteristics as suggested by the TIDieR-PHP template [22]. MCMV was implemented in July 2009 by the Brazilian Federal Government [23]. Its main goals are to reduce the housing shortage in Brazil (which exceeded six million houses in 2016), with 89% of unmet need concentrated among low-income families (defined as earning less than three times the minimum wage), and improve the construction sector through job

generation and wider Brazilian economic growth [23]. By 2018, the government had contracted 5,164,075 and delivered 3,787,200 million house units, resulting in over six million people receiving housing across Brazil [24].

MCMV was structured to reach families from different income classes using three distinct eligibility criteria and subsidies. In this study, we focus on Class 1 subsidies, which targets low-income families, defined as households with less than three times the minimum wage (622,0 BRL in 2010, equivalent to USD 116,25) per month. Class 1 subsidies are divided in two subprogrammes targeting either urban or rural areas. In municipalities with more than 50 thousand inhabitants, the MCMV programme uses the Residential Lease Fund (FAR-MCMV) to build or acquire new housing units. Individuals eligible for Class 1 MCMV living in municipalities with less than 50,000 inhabitants or living in rural areas receive other forms of MCMV adapted to their context. For this study, we focus on FAR-MCMV, which is the largest subprogramme of MCMV. From 2009 to 2015, we estimate that FAR-MCMV delivered over 1.2 million house units across the country [24].

Eligibility criteria for FAR-MCMV include: *i)* the applicant must have a household income less than or equal to three times the minimum wage (without considering other social benefits, such as income from a conditional cash transfer program); *ii)* not be an owner, assignee or promising buyer of a residential property; and *iii)* not have received any previous housing benefits or grants for the purchase of construction materials [23]. Priority criteria for FAR-MCMV include: *i)* living in a hazardous or unhealthy area or being homeless; *ii)* belonging to a family headed by a lone mother (i.e. no male partner); *iii)* having a disabled person(s) in the household, with legal proof; and *iv)* having elderly people, aged 60 years old or over within the household [23].

Logic models

We created a logic model for each of the health outcomes studied in this protocol, informed by the existing literature, to describe the hypothesised mechanisms through which the MCMV may affect (a) CVD mortality, (b) leprosy new case detection (here and after, named as “incidence”), and (c) tuberculosis incidence. We identified, for each of the outcomes, pathways that are likely to operate through direct (physical house conditions) and indirect forms (housing neighbourhood effects and subjective aspects associated with house ownership).

a) Logic model for CVD mortality

The Programme may affect cardiovascular health through different pathways. Possible direct effects include changes in the physical standards of housing, leading to improvements in thermal and acoustic comfort, basic sanitation and household density reduction [2,25–27]. Indirect effects are related to the inclusion of families in new social and physical neighbourhood environments – due to the relocation process – with potentially better socio-economic, structural and physical contexts, as well as better access to basic health services [9,28,29]. On the other hand, relocation may negatively influence the social environment of the neighbourhood, since beneficiaries lose social networks and support, reducing social cohesion [3,11,30].

In the short term, improvements in living conditions could enable changes in health behaviours, like physical activity, diet, alcohol and tobacco consumption [4,10]. In addition, reducing the cost of housing also provides greater access to resources which can be spent on healthier food and health care, leading to better control of cardiometabolic risk factors [31,32].

In the long-term, the Programme could contribute to reducing chronic and cumulative exposure to psychosocial risk factors arising from inadequate housing contexts and this could potentially reduce the incidence of cardiovascular events [33,34]. The effects of the Programme on cardiovascular outcomes might be differential by age, race/ethnicity, socioeconomic position and gender, and might contribute to the reduction of disparities in cardiovascular mortality in Brazil.

Figure 1: Logic model to evaluate the effect of Minha Casa Minha Vida on reduction of cardiovascular mortality (Appendix I).

b) Logic model for leprosy

There is a strong relationship between housing and differential leprosy exposure [35–38]. Housing with better infrastructure and access to drinking water and adequate sanitation may improve hygiene conditions [17,39]. Reduction in household crowding leads to lower contacts among members of the family and, consequently, reduction of leprosy transmission [40,41].

Access to neighbourhood amenities might improve access to health services, as well as improve community cohesion, which are important influences on leprosy risk [35,42]. Places to purchase healthy food can facilitate food security, which might result in improvements in the nutritional status of individuals and accelerate the immune response in leprosy infection [35,43].

Housing ownership may give feelings of security and/or prestige. Also, it can provide greater availability of income for expenditure on potentially health-enhancing products such as food and health care [12,44–46]. It is known that access to health care is important to support strategies for self-care, case detection, timely diagnosis and treatment, and prevention of more severe forms of the disease [47,48].

Figure 2: Logic model to evaluate the effect of Minha Casa Minha Vida on Leprosy Incidence (Appendix I).

c) Logic model for tuberculosis

The Programme may affect tuberculosis incidence through different pathways. Better housing leads to better ventilation and overall housing conditions, such as exposure to sunlight and reduced crowding, which reduce the possibility of transmission through aerial dispersion of the bacillus [49,50]. Alternatively, we may observe beneficiaries experiencing better quality of life or socioeconomic status due to a better environment [50]. Better housing location, if accompanied by more access to public services, including health care, can alter individual susceptibility to disease and improve treatment outcomes for those already infected. On the other hand, longer distances to school and workplace may lead to physical stress, worsened immunity and predispose people to infection given exposure to *Mycobacterium tuberculosis* (MTB) [19,51]. Social support may also change due to the relocation, and may affect exposure to the agent as well as disease progression [52].

We expect a differential effect of this housing programme by age group as the direct effects of the housing material will be more pronounced in children, especially in the case of intradomiciliary contacts of tuberculosis cases. As people grow older and become more exposed to external environment related factors, we hypothesise the effect of the programme to be the net effect of improved housing quality and surrounding area related factors.

Figure 3: Logic model to evaluate the effect of Minha Casa Minha Vida on tuberculosis incidence (Appendix I).

Datasets

We will link deterministically the baseline of the 100 Million Brazilian Cohort (2001-2015), which includes information on socioeconomic and demographic variables, to the FAR-MCMV (2009-2015), the Mortality Information System (SIM) (2007-2015), and the leprosy (2007-2015) and tuberculosis (2007-2015) registries from the Notifiable Disease Information System [21,53,54]. The final deidentified dataset will contain information from recipients and non-recipients of the FAR-MCMV.

Sociodemographic variables

The 100 Million Brazilian Cohort includes baseline socioeconomic and demographic data from over 114 million individuals (approximately 55% of the Brazilian population in 2019). The cohort comprises people who are enrolled in the *Cadastro Unico*, a register containing all individuals within a household that have applied for any social programme administered by the Brazilian Federal Government [21]. We extracted individual level information (age, sex, race/ethnicity, education and occupation), household characteristics (household density, region and area of residence, household construction material, water supply, sanitation, electricity and waste disposal); and monthly per capita income for all family members [21].

Minha Casa Minha Vida Programme – FAR modality

Socioeconomic and demographic information (date of birth, gender, marital status, household monthly per capita income, name of head of household) from those who sign the contract to receive the housing unit – the main beneficiary – is obtained from the FAR-MCMV database [21]. In addition, we will extract from this database the name and Social identification number (SIN) from those who sign the contract, the date of signature of the contract (i.e., a *proxy* for the time that housing unit was delivered to the family), individuals who live with the main beneficiary at that time, and the address of the housing unit or the condominium delivered [21].

Mortality Information System (SIM)

Deaths within Brazil are subject to certification by medical professionals, so the causes of death (using ICD-10 codes) can be ascertained reasonably precisely. Despite the death registry being compulsory, there is evidence of under-ascertainment of deaths,

particularly within areas that are more rural and in poorest regions of Brazil [55]. Coding of CVD related causes is thought to be good, but for some analyses, corrections are needed to take account of ill-defined causes [56]. In addition, since 2010 the proportion of garbage codes, causes of deaths that should not be considered as underlying causes of deaths, had declined, and in 2015, an estimation, show that 97.2% of deaths were included in the mortality system [56]. The highest ascertainment was observed in the state of São Paulo (99.8%), located in the Southeast region, and the lowest was observed in the state of Amapá (91.2%), in the North region of the country [56]. Under-ascertainment and miscoding of deaths is known to be most problematic in older adults, as well in young children [56].

Notifiable Diseases Information System (SINAN)

Leprosy and tuberculosis notification to the SINAN disease registry is compulsory in Brazil and coded using ICD-10. The records include individuals' socio-demographic and clinical information at the time of diagnosis and treatment updates when available. SINAN has improved its quality and completeness over time [57]. However, underreported cases and missing information still happen, especially in the poorest regions of the country. Therefore, leprosy and tuberculosis reporting to the SINAN notification system is based on passive surveillance and there is therefore heterogeneity in the frequency and completeness of reporting, which may result in the true incidence of diseases being underestimated [57].

Data analysis plan

Definitions of exposure and outcomes

We will define our exposed population as individuals who signed the contract to receive the housing unit from FAR-MCMV and the household members who live with them at the time of the contract signature. If there are no household members registered at the time of contract signature, we will include household members that appear in the last update of *Cadastro Único* carried out up to 2 years before receipt of the house unit from FAR-MCMV.

All outcomes will be defined according to the International Classification of Diseases, version 10 (ICD-10). We will evaluate the following outcomes: *i)* all-cause mortality; *ii)* CVD mortality (I00-I99); *iii)* ischaemic heart disease mortality (I20-I25); iv) cerebrovascular disease mortality (I60-I69); *v)* incidence of leprosy (A30); and *vi)* incidence of tuberculosis (A15-A19). All-cause and CVD mortality will be evaluated in adults aged 30 to 69 years old

(defined as premature mortality within Brazil and of particular relevance to health policy) and in adults aged 18 to 69 years old (the broader adult population with the most reliable mortality data). We will not investigate outcomes among older adults, given the known issues of ascertaining mortality for this age group within the mortality information system. The incidence of leprosy and tuberculosis will be evaluated in all age groups.

To evaluate all-cause and specific cardiovascular mortality in individuals aged 18-69 years old, the follow-up time (in years) for each individual will start at entry into the analytical cohort (i.e. on signing a contract for the intervention group or the matching date for the control group) or the age at which an individual reaches 18 years of age, whichever is later. The follow-up time will end at the first of: date of death, end of follow-up (31st December 2015) or reaching 70 years of age.

To evaluate the incidence of leprosy and tuberculosis among individuals for all ages, the follow-up time (in years) for each individual will start at entry into the analytical cohort and will end at the first diagnosis of leprosy or tuberculosis, date of death or end of follow-up (31st December 2015).

Analysis

Estimating the effect of the FAR-MCMV programme on health outcomes is challenging due to selective uptake of the intervention by individuals. To address this, we will use different propensity score (PS) approaches to identify comparable individuals (based on observable characteristics) who did and did not receive the FAR-MCMV intervention, given individual-level characteristics [58]. Matching methods will include nearest neighbour matching using narrow callipers to minimise bias and Kernel matching [59,60]. In addition to matching methods, we will also estimate the effect of MCMV on the selected outcome using survival models weighted by the Inverse Probability of Treatment (IPTW) to estimate the average treatment effects (ATEs) and average treatment effect on the treated (ATTs) [58].

In addition, we will stratify the analyses for key subgroups of interest – namely, gender, race/ethnicity, socioeconomic position and phases of implementation of MCMV programme. We will also investigate whether effects differ across combinations of these characteristics. Specifically for leprosy, we will also stratify the analyses by residence in a high leprosy burden municipality as defined by the Brazilian Ministry of Health [61]. We will

For peer review only - http://bmjopen.bmj.com/site/about/guidelines.xhtml

also stratify our analyses for individuals that are (and are not) beneficiaries of the *Bolsa Familia Programme* (BFP), one of the largest conditional cash transfer of the world [62].

Robustness checks

To check the robustness of our findings we will perform different tests. First, in the propensity score matching analysis, we will restrict our data from matching individuals with more narrow PS (different callipers). We will also restrict our analysis to certain types of municipality where data from MCMV-FAR or the mortality information system have better quality.

Sensitivity analyses

The matching strategy relies on the outcome being independent of treatment, conditional on the propensity score (Conditional Independence Assumption – CIA) [63]. However, if there are unobserved variables which affect assignment into treatment and the outcome variable simultaneously, a bias might arise. Since this assumption is non-testable by its nature, questions about the plausibility of the CIA can arise, and our results or at least their statistical significance could probably be driven by an omitted variable strongly correlated with the treatment outcome.

We will carry out sensitivity analyses to assess how strong the influence of these postulated unobservables might be in our study. We will use Rosenbaum bounds approach [64] and the sensitivity strategy proposed by Ichino et al [63]. This approach aims to assess the bias of our estimates when the CIA is assumed to fail in some meaningful way. A failure in the CIA is equivalent to saying that the assignment to treatment is not unconfounded given the set of observable variables [63].

Ethics and dissemination

The 100 Million Brazilian Cohort study was approved by the ethics committee of *Instituto Gonçalo Muniz – Oswaldo Cruz Foundation* (project number: 1.612.302) and the specific aims of this project was submitted for ethical approval in the same ethics committee. In addition, the University of Glasgow Medical, Veterinary & Life Sciences College Ethics Committee also approved the study (project number: 200190001). All data are linked in a safe room with access restricted to specified people only. After the data are linked and the linkage accuracy is calculated, researchers will have full access to the deidentified dataset. The dataset

will be accessed by researchers upon application to a data curation committee with a detailed analysis plan. The dataset will receive a Digital Object Identifier (DOI), and full specification of how the dataset was created will be available online. All manuscripts will be published in in high quality peer-reviewed open access journals and will also be disseminated to policy makers through stakeholder events and policy briefs.

Authors' contributions: SVK, AJFF, JMP wrote the first draft of the protocol. RR, RFO, CSST, MNS, RLF, MYT, RO, EMLA, SA, PC, LS, AHL and MLB contributed with additional material. All authors reviewed the final version.

Funding statement: This research was funded by the National Institute for Health Research (NIHR) (GHRG /16/137/99) using UK aid from the UK Government to support global health research. The Social and Public Health Sciences Unit is core funded by the Medical Research Council (MC_UU_12017/13) and the Scottish Government Chief Scientist Office (SPHSU13). SVK is funded by a NHS Research Scotland Senior Clinical Fellowship (SCAF/15/02). Cidacs/Fiocruz is supported by grants from CNPq/MS/Gates Foundation (401739/2015-5), the Wellcome Trust, UK (202912/Z/16/Z) and the Health Surveillance Secretariat, Ministry of Health, Brazil. The views expressed in this publication are those of the authors and not necessarily those of the NIHR, the UK Department of Health and Social Care or the Ministry of Heath, Brazil.

Competing interest's statement: The authors declare no competing interests.

References

1 World Health Organization. *WHO housing and health guidelines.* 2018.

2 Braubach M, Jacobs DE, Ormandy D. Environmental burden of disease associated with inadequate housing. 2011.

3 Barber S, Hickson DA, Wang X, *et al.* Neighborhood disadvantage, poor social conditions, and cardiovascular disease incidence among African American adults in the Jackson heart study. *Am J Public Health* 2016;**106**:2219–26. doi:10.2105/AJPH.2016.303471

4 Wang X, Auchincloss AH, Barber S, *et al.* Neighborhood social environment as risk factors to health behavior among African Americans: The Jackson Heart Study. *Heal Place* 2017;**45**:199–207. doi:10.1016/j.healthplace.2017.04.002

5 Thomson H, Thomas S. Developing empirically supported theories of change for housing investment and health. *Soc Sci Med* 2015;**124**:205–14. doi:10.1016/j.socscimed.2014.11.043

6 Krieger J, Higgins DL. Housing and health: time again for public health action. *Am J Public Health* 2002;**92**:758–68.http://www.ncbi.nlm.nih.gov/pubmed/11988443%0Ahttp://www.pubmedcentral.nih.gov/art

iclerender.fcgi?artid=PMC1447157

7 Evans GW, Wells NM, Moch A. Housing and Mental Health: A Review of the Evidence and a Methodological and Conceptual Critique LK - https://tue.on.worldcat.org/oclc/437737953. *J Soc Issues TA - TT* - 2003;**59**:475–500.

8 Diez Roux A V. Neighborhoods and Health: What Do We Know? What Should We Do? *Am J Public Health* 2016;**106**:430–1. doi:10.2105/AJPH.2016.303064

9 Malambo P, Kengne AP, De Villiers A, *et al.* Built Environment, Selected Risk Factors and Major Cardiovascular Disease Outcomes: A Systematic Review. *PLoS One* 2016;**11**:e0166846. doi:10.1371/journal.pone.0166846

10 Poelman M, Strak M, Schmitz O, *et al.* Relations between the residential fast-food environment and the individual risk of cardiovascular diseases in The Netherlands: A nationwide follow-up study. *Eur J Prev Cardiol* 2018;**25**:1397–405. doi:10.1177/2047487318769458

11 Robinette JW, Charles ST, Gruenewald TL. Neighborhood cohesion, neighborhood disorder, and cardiometabolic risk. *Soc Sci Med* 2018;**198**:70–6. doi:10.1016/j.socscimed.2017.12.025

12 Bray N, Burns P, Jones A, *et al.* Costs and outcomes of improving population health through better social housing: a cohort study and economic analysis. *Int J Public Health* 2017;**62**:1039–50. doi:10.1007/s00038-017-0989-y

13 Brasil. Ministério da Saúde. Sala de Apoio à Gestão Estratégica do Ministério da Saúde (SAGE). Indicadores de morbidade: Hanseníase. Ministério da Saúde. 2019.http://sage.saude.gov.br/#

14 Brasil. Sala de apoio a gestão estratégica. Situação de saúde. Indicadores de morbidade: Tuberculose. Ministério da Saúde. 2019.https://sage.saude.gov.br/ (accessed 25 May 2020).

15 WHO. World Health Organization. Cardiovascular disease: prevention and control. 2009.http://www.who.int/dietphysicalactivity/publications/facts/cvd/en/

16 Brasil. Saúde Brasil 2018. 2018. http://bvsms.saude.gov.br/bvs/publicacoes/saude_brasil_2018_analise_situacao_saude_doencas_agravos_cronicos_desafios_perspectivas.pdf (accessed 15 Oct 2019).

17 Pescarini JM, Strina A, Nery JS, *et al.* Socioeconomic risk markers of leprosy in high-burden countries: A systematic review and meta-analysis. *PLoS Negl Trop Dis* 2018;**12**:1–20. doi:10.1371/journal.pntd.0006622

18 Dawson P, Perri BR, Ahuja SD. High Tuberculosis Strain Diversity Among New York City Public Housing Residents. *Am J Public Health* 2016;**106**:563–8. doi:10.2105/AJPH.2015.302910

19 Floyd K, Glaziou P, Zumla A, *et al.* The global tuberculosis epidemic and progress in care, prevention, and research: an overview in year 3 of the End TB era. Lancet Respir. Med. 2018;**6**:299–314. doi:10.1016/S2213-2600(18)30057-2

20 Centro de Integração de Dados e Conhecimento para a Saúde. The 100 Million Brazilian Cohort. 2019.https://cidacs.bahia.fiocruz.br/

21 Barreto ML, Almeida BDA, Ichihara MY, *et al.* The Center for Data and Knowledge Integration for Health (CIDACS). *Int J Popul Data Sci* 2019;**4**:1–24. doi:10.23889/ijpds.v4i2.1140

22 Campbell M, Katikireddi SV, Hoffmann T, *et al.* TIDieR-PHP: A reporting guideline for population health and policy interventions. *BMJ* 2018;**361**:1–5. doi:10.1136/bmj.k1079

23 Brasil. Lei N° 11.977, de 7 de julho de 2009. Regulamenta o Programa Minha Casa Minha

Vida. Brasil.: : http://www.planalto.gov.br/ccivil_03/_Ato2007-2010/2009/Lei/L11977.htm 2009. http://www.planalto.gov.br/ccivil_03/_Ato2007-2010/2009/Lei/L11977.htm (accessed 1 Sep 2019).

24 Brasil. Boletim mensal sobre os subsidios da União: Programa Minha Casa Minha Vida (10ª Edição). Brasília, Distrito Federal: 2019.

25 Gemmell I. Indoor heating, house conditions, and health. *J Epidemiol Community Heal* 2001;**55**:928–9. doi:10.1136/jech.55.12.928

26 Smith KR. Indoor air pollution in developing countries: recommendations for research. *Indoor Ari* 2003;**12**:198–207.

27 Sørensen M, Andersen ZJ, Nordsborg RB, *et al.* Road traffic noise and incident myocardial infarction: A prospective cohort study. *PLoS One* 2012;**7**:1–7. doi:10.1371/journal.pone.0039283

28 Diez Roux A V, Borrell LN, Haan M, *et al.* Neighbourhood environments and mortality in an elderly cohort: Results from the cardiovascular health study. *J Epidemiol Community Health* 2004;**58**:917–23. doi:10.1136/jech.2003.019596

29 Brown AF, Liang L-JJ, Vassar SD, *et al.* Neighborhood socioeconomic disadvantage and mortality after stroke. *Neurology* 2013;**80**:520–7. doi:10.1212/WNL.0b013e31828154ae

30 Chaix B, Lindstrom M, Rosvall M, *et al.* Neighbourhood social interactions and risk of acute myocardial infarction. *J Epidemiol Community Heal* 2008;**62**:62–8. doi:10.1136/jech.2006.056960

31 Chambers EC, Hanna DB, Hua S, *et al.* Relationship between area mortgage foreclosures, homeownership, and cardiovascular disease risk factors: The Hispanic Community Health Study/Study of Latinos. *BMC Public Health* 2019;**19**:1–8. doi:10.1186/s12889-019-6412-2

32 Chambers EC, Rosenbaum E. Cardiovascular health outcomes of latinos in the affordable housing as an obesity mediating environment (AHOME) study: A study of rental assistance use. *J Urban Heal* 2014;**91**:489–98. doi:10.1007/s11524-013-9840-9

33 Robinette JW, Charles ST, Almeida DM, *et al.* Neighborhood features and physiological risk: An examination of allostatic load. *Heal Place* 2016;**41**:110–8. doi:10.1016/j.healthplace.2016.08.003

34 Erqou S, Echouffo-Tcheugui JB, Kip KE, *et al.* Association of cumulative social risk with mortality and adverse cardiovascular disease outcomes. *BMC Cardiovasc Disord* 2017;**17**:1–11. doi:10.1186/s12872-017-0539-9

35 Kerr-Pontes LRS, Barreto ML, Evangelista CMN, *et al.* Socioeconomic, environmental, and behavioural risk factors for leprosy in North-east Brazil: Results of a case-control study. *Int J Epidemiol* 2006;**35**:994–1000. doi:10.1093/ije/dyl072

36 Murto C, Chammartin F, Schwarz K, *et al.* Patterns of Migration and Risks Associated with Leprosy among Migrants in Maranhão, Brazil. *PLoS Negl Trop Dis* 2013;**7**. doi:10.1371/journal.pntd.0002422

37 Kumar A, Girdhar A, Yadav VS, *et al.* Some Epidemiological Observations on Leprosy in India. *Int J Lepr* 2001;**69**:234–40.

38 Nery JS, Ramond A, Pescarini JM, *et al.* Socioeconomic determinants of leprosy new case detection in the 100 Million Brazilian Cohort: a population-based linkage study. *Lancet Glob Heal* Published Online First: 2019. doi:10.1016/S2214-109X(19)30260-8

39 WHO/UNICEF. Progress on Sanitation and Drinking Water: 2015 Update and MDG Assessment. 2015. doi:10.1007/s13398-014-0173-7.2

40 Fine PEM, Sterne JAC, Pönnighaus JM, *et al.* Household and dwelling contact as risk factors for leprosy in northern Malawi. *Am J Epidemiol* 1997;**146**:91–102. doi:10.1093/oxfordjournals.aje.a009195

41 Moet FJ, Pahan D, Schuring RP, *et al.* Physical Distance, Genetic Relationship, Age, and Leprosy Classification Are Independent Risk Factors for Leprosy in Contacts of Patients with Leprosy. *J Infect Dis* 2006;**193**:346–53. doi:10.1086/499278

42 Van Beers SM, De Wit MYL, Klatser PR. The epidemiology of mycobacterium leprae: Recent insight. *FEMS Microbiol Lett* 1996;**136**:221–30. doi:10.1016/0378-1097(95)00505-6

43 Teixeira CSS, de Medeiros DS, Alencar CH, *et al.* Nutritional aspects of people affected by leprosy, between 2001 and 2014, in semi-arid Brazilian municipalities. *Cienc e Saude Coletiva* 2019;**24**:2431–41. doi:10.1590/1413-81232018247.19642017

44 Howden-Chapman P, Chapman R. Health co-benefits from housing-related policies. *Curr Opin Environ Sustain* 2012;**4**:414–9. doi:10.1016/j.cosust.2012.08.010

45 Baker E, Mason K, Bentley R, *et al.* Exploring the Bi-directional Relationship between Health and Housing in Australia. *Urban Policy Res* 2014;**32**:71–84. doi:10.1080/08111146.2013.831759

46 Lindberg RA, Shenassa ED, Acevedo-Garcia D, *et al.* Housing interventions at the neighborhood level and health: a review of the evidence. *J Public Health Manag Pract* 2010;**16**:44–52. doi:10.1097/PHH.0b013e3181dfbb72

47 Barreto ML, Teixeira MG, Bastos FI, *et al.* Successes and failures in the control of infectious diseases in Brazil: Social and environmental context, policies, interventions, and research needs. Lancet. 2011;**377**:1877–89. doi:10.1016/S0140-6736(11)60202-X

48 Moschioni C, Antunes CM de F, Grossi MAF, *et al.* Fatores de risco para incapacidade física no momento do diagnóstico de 19.283 casos novos de hanseníase. *Rev Soc Bras Med Trop* 2010;**43**:19–22. doi:10.1590/S0037-86822010000100005

49 Wingfield T, Schumacher SG, Sandhu G, *et al.* The seasonality of tuberculosis, sunlight, vitamin D, and household crowding. *J Infect Dis* 2014;**210**:774–83. doi:10.1093/infdis/jiu121

50 Hargreaves JR, Boccia D, Evans CA, *et al.* The social determinants of tuberculosis: from evidence to action. *Am J Public Health* 2011;**101**:654–62. doi:10.2105/AJPH.2010.199505

51 Hayward SE, Dowd JB, Fletcher H, *et al.* A systematic review of the impact of psychosocial factors on immunity: Implications for enhancing BCG response against tuberculosis. *SSM - Popul Heal* 2020;**10**:100522. doi:10.1016/j.ssmph.2019.100522

52 Paz-Soldan VA, Alban RE, Jones CD, *et al.* The provision of and need for social support among adult and pediatric patients with tuberculosis in Lima, Peru: a qualitative study. *BMC Health Serv Res* 2013;**13**:290.

53 Sanni Ali M, Ichihara MY, Lopes LC, *et al.* Administrative data linkage in Brazil: Potentials for health technology assessment. *Front Pharmacol* 2019;**10**:1–20. doi:10.3389/fphar.2019.00984

54 Pita R, Pinto C, Sena S, *et al.* On the Accuracy and Scalability of Probabilistic Data Linkage over the Brazilian 114 Million Cohort. *IEEE J Biomed Heal Informatics* 2018;**22**:346–53. doi:10.1109/JBHI.2018.2796941

55 Franc¸a E, Teixeira R, Ishitani L, *et al.* Ill-defined causes of death in Brazil: A redistribution method based on the investigation of such causes. *Rev Saude Publica* 2014;**48**:671–81. doi:10.1590/S0034-8910.2014048005146

56 IBGE. Instituto Brasileiro de Geografia e Estatística. Informação Demográfica e

Socioeconômica. Sistemas de estatísticas vitais no Brasil: avanços, perspectivas e desafios. 2018. https://biblioteca.ibge.gov.br/visualizacao/livros/liv101575.pdf

57 Brasil. Ministério da Saúde. Secretaria de Vigilância à Saúde. Guia de Vigilância Epidemiológica. 2005. http://portal.anvisa.gov.br/documents/33916/388729/Guia_Vig_Epid_novo2.pdf/99464018-d6d1-486b-853b-9871d6eff16f?version=1.0

58 Austin PC. The performance of different propensity score methods for estimating marginal hazard ratios. *Stat Med* 2013;**32**:2837–49. doi:10.1002/sim.5705

59 Lunt M. Selecting an appropriate caliper can be essential for achieving good balance with propensity score matching. *Am J Epidemiol* 2014;**179**:226–35. doi:10.1093/aje/kwt212

60 Baser O. Too much ado about propensity score models? Comparing methods of propensity score matching. *Value Heal* 2006;**9**:377–85. doi:10.1111/j.1524-4733.2006.00130.x

61 Brasil. Ministério da Saúde. Sistema de Legislação da Saúde. Portaria nº 2.556, de 28 de outubro de 2011. Avaiable from:

62 Soares FV, Ribas RP, Osório RG. Evaluating the impact of Brazil's Bolsa Família: Cash transfer programs in comparative perspective. *Lat Am Res Rev* 2010;**45**:173–90.http://www.undp-povertycentre.org

63 Ichino A, Mealli F, Nannicini T. From temporary helps jobs to permanent employment: what can we learn from matching estimators and their sensitivity? *J Appl Econom* 2008;**23**:305–27. doi:10.1002/jae

64 Rosenbaum P. *Observational Studies*. New York: : Springer US 2002.

Evaluating the health effect of a Social Housing programme, Minha Casa Minha Vida, using the 100 million Brazilian Cohort: A natural experiment study protocol

1. Appendix: Logic models

Figure 1: Logic model to evaluate the effect of Minha Casa Minha Vida on reduction of Cardiovascular mortality.

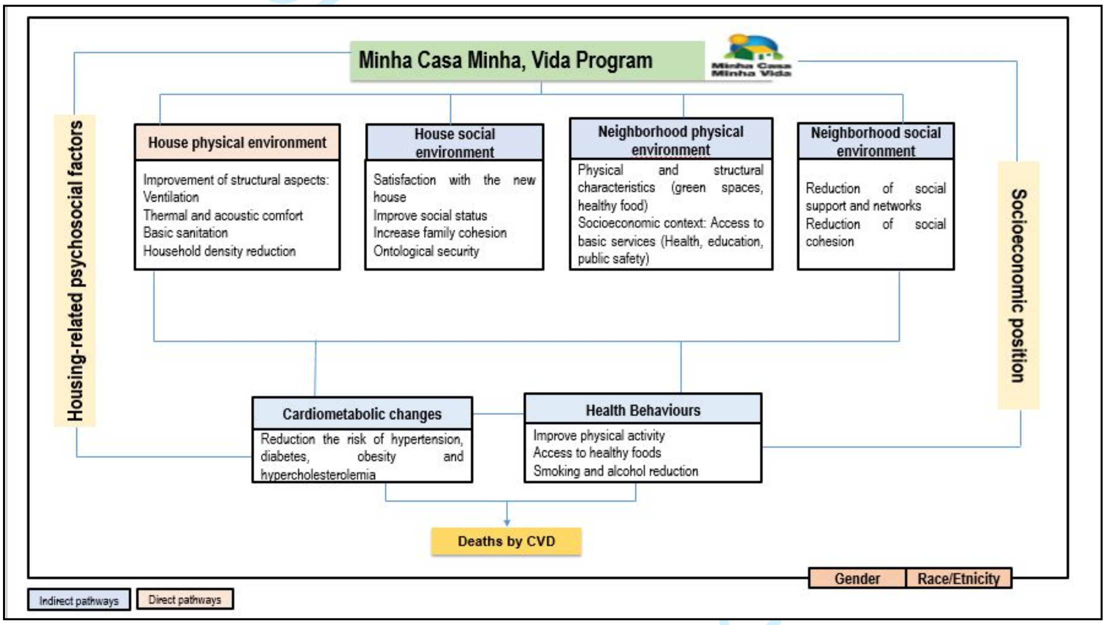

For peer review only

Figure 2: Logic model to evaluate the effect of *Minha Casa Minha Vida* on leprosy incidence.

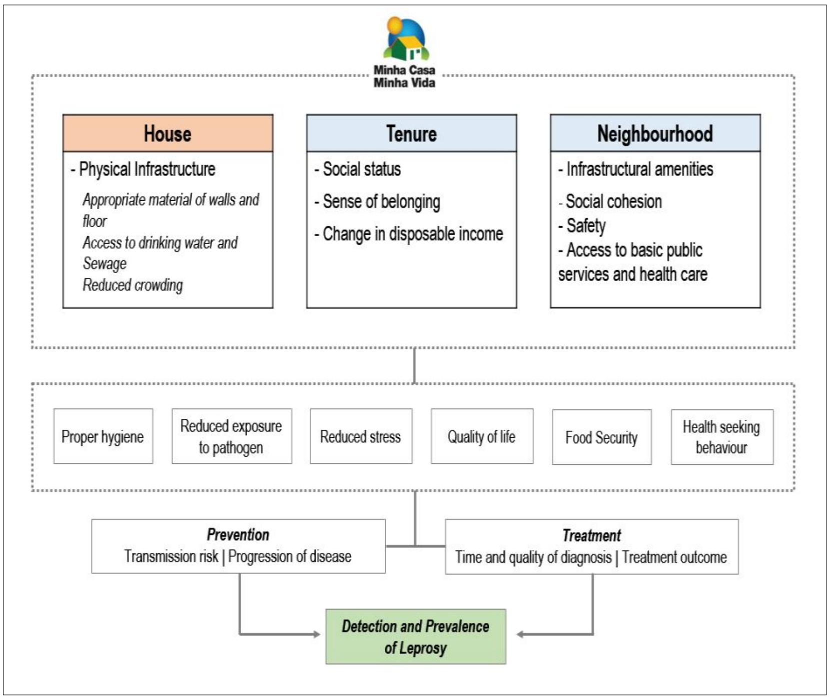

Figure 3: Logic model to evaluate the effect of Minha Casa Minha Vida on Tuberculosis Incidence.

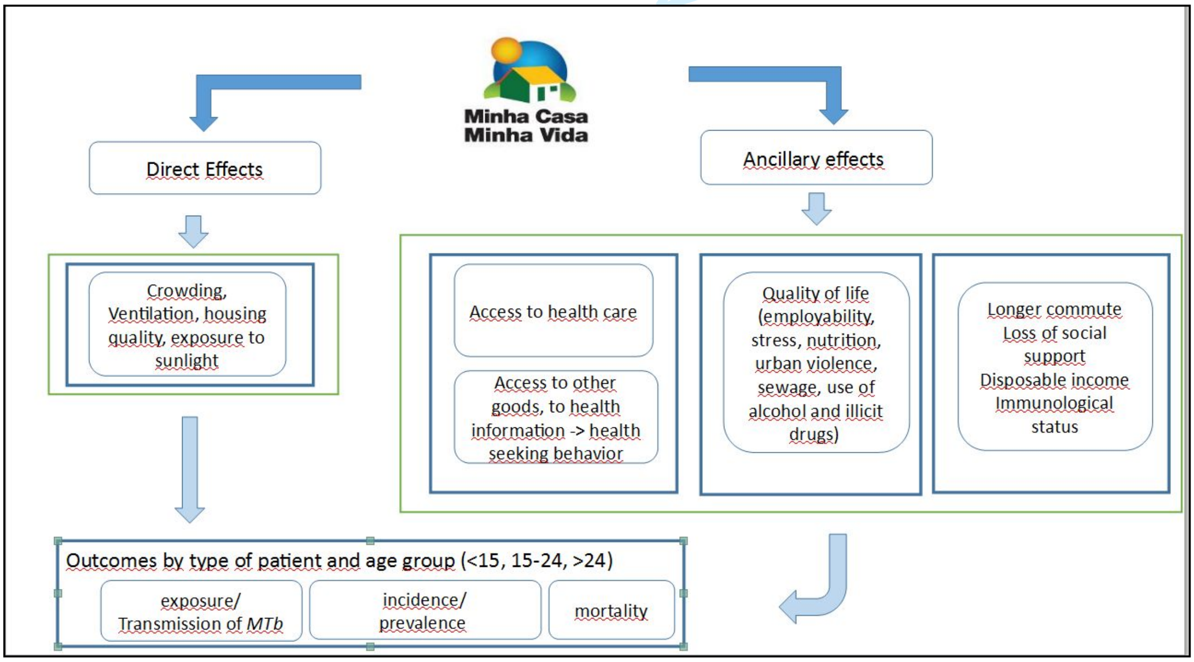

For peer review only - http://bmjopen.bmj.com/site/about/guidelines.xhtml

BMJ Open

Evaluating the health effect of a Social Housing programme, Minha Casa Minha Vida, using the 100 million Brazilian Cohort: A natural experiment study protocol

Journal:	*BMJ Open*
Manuscript ID	bmjopen-2020-041722.R1
Article Type:	Protocol
Date Submitted by the Author:	03-Dec-2020
Complete List of Authors:	Ferreira, Andrêa; Federal University of Bahia, Public Health Institute; Fiocruz Bahia, Centro de Integração de Dados e Conhecimentos para Saúde (Cidacs)<br>Pescarini, Julia; Fundação Oswaldo Cruz, Centro de Integração de Dados e Conhecimentos para Saúde (Cidacs)<br>Sanchez, Mauro; Universidade de Brasília, Public Health<br>Flores-Ortiz, Renzo; Fiocruz Bahia, Center for Integration of Data and Health Knowledge (Cidacs)<br>Teixeira , Camila ; Universidade Federal da Bahia, Public Health Institute<br>Fiaccone, Rosemeire; Universidade Federal da Bahia, Mathematics and Statistics<br>Ichihara, Maria Yury; Universidade Federal da Bahia, Public Health Institute<br>Oliveira , Rodrigo; Federal University of Bahia, Economy<br>Aquino, Estela M. L.; Universidade Federal da Bahia, Public Health Institute<br>Smeeth, Liam; London School of Hygiene and Tropical Medicine Faculty of Public Health and Policy, Epidemiology and Population Health<br>Craig , Peter ; University of Glasgow MRC/CSO Social and Public Health Sciences Unit, Public Health Sciences Unit<br>Ali, Sanni; London School of Hygiene & Tropical Medicine<br>Leyland, Alastair; University of Glasgow MRC/CSO Social and Public Health Sciences Unit, Public Health Sciences Unit<br>Barreto, Mauricio; Universidade Federal da Bahia, Public Health Institute<br>Ribeiro , Rita de Cássia; Universidade Federal da Bahia, Public Health Institute<br>Katikireddi, Srinivasa; University of Glasgow School of Life Sciences, MRC/CSO Social & Public Health Sciences Unit
**Primary Subject Heading**:	Public health
Secondary Subject Heading:	Cardiovascular medicine, Health policy, Infectious diseases, Epidemiology
Keywords:	Coronary heart disease < CARDIOLOGY, Tuberculosis < INFECTIOUS DISEASES, Tropical medicine < INFECTIOUS DISEASES, PUBLIC HEALTH, EPIDEMIOLOGY

I, the Submitting Author has the right to grant and does grant on behalf of all authors of the Work (as defined in the below author licence), an exclusive licence and/or a non-exclusive licence for contributions from authors who are: i) UK Crown employees; ii) where BMJ has agreed a CC-BY licence shall apply, and/or iii) in accordance with the terms applicable for US Federal Government officers or employees acting as part of their official duties; on a worldwide, perpetual, irrevocable, royalty-free basis to BMJ Publishing Group Ltd ("BMJ") its licensees and where the relevant Journal is co-owned by BMJ to the co-owners of the Journal, to publish the Work in this journal and any other BMJ products and to exploit all rights, as set out in our licence.

The Submitting Author accepts and understands that any supply made under these terms is made by BMJ to the Submitting Author unless you are acting as an employee on behalf of your employer or a postgraduate student of an affiliated institution which is paying any applicable article publishing charge ("APC") for Open Access articles. Where the Submitting Author wishes to make the Work available on an Open Access basis (and intends to pay the relevant APC), the terms of reuse of such Open Access shall be governed by a Creative Commons licence – details of these licences and which Creative Commons licence will apply to this Work are set out in our licence referred to above.

Other than as permitted in any relevant BMJ Author's Self Archiving Policies, I confirm this Work has not been accepted for publication elsewhere, is not being considered for publication elsewhere and does not duplicate material already published. I confirm all authors consent to publication of this Work and authorise the granting of this licence.

For peer review only

Evaluating the health effect of a Social Housing programme, Minha Casa Minha Vida, using the 100 million Brazilian Cohort: A natural experiment study protocol

Andrêa JF Ferreira[1,2*], Julia M Pescarini[1], Mauro N Sanchez[1,3,] Renzo Flores-Ortiz[1], Camila SS Teixeira[1,2], Rosemeire L Fiaccone[1,4,] Maria YT Ichihara[1], Rodrigo Oliveira[1,5], Estela ML Aquino[1,2], Liam Smeeth[6,7], Peter Craig[8], Sanni Ali[6], Alastair H Leyland[8], Mauricio L Barreto[1,2], Rita de Cássia Ribeiro Silva[1,2**], Srinivasa Vittal Katikireddi[8**].

[1] Center for Integration of Data and Health Knowledge (Cidacs), Gonçalo Muniz Institute (Fiocruz/Bahia).

[2] Institute of Public Health, Federal University of Bahia, Salvador, Brazil.

[3] Brazilian University (UnB), Brasilia, Brazil.

[4] Institute of Mathematics and Statistics, Universidade Federal da Bahia, Salvador, Brazil.

[5] Department of Economics, Federal University of Bahia, Salvador, Bahia.

[6] Department of Non-Communicable Disease Epidemiology, London School of Hygiene and Tropical Medicine (LSHTM), London, United Kingdom.

[7] Health Data Research (HDR), London, United Kingdom.

[8] MRC/CSO Social and Public Health Sciences Unit, University of Glasgow, Glasgow, United Kingdom.

***Corresponding Author:** Andrêa JF Ferreira

Email: andreaferreiracv@gmail.com

Postal address: Centro de Integração de Dados e Conhecimentos para Saúde (Cidacs), Fundação Oswaldo Cruz. Edf. Tecnocentro – R. Mundo, 121 – Trobogy, Salvador – Bahia/Brazil. Postal code: 41745-715.

****Co-senior authors**

Keywords: Cardiovascular diseases, leprosy, tuberculosis, social housing, social protection, social policies, natural experiment.

Abstract

Introduction

Social housing programmes have been shown to influence health, but their effects on cardiovascular mortality and incidence of infectious diseases, such as leprosy and tuberculosis, are unknown. We will use individual administrative data to evaluate the effect of the Brazilian housing programme *Minha Casa Minha Vida* (MCMV) on cardiovascular disease mortality and incidence of leprosy and tuberculosis.

Methods and analysis

We will link the baseline of the 100 Million Brazilian Cohort (2001-2015), which includes information on socioeconomic and demographic variables, to the MCMV (2009-2015), CVD mortality (2007-2015), leprosy (2007-2015), and tuberculosis (2007-2015) registries. We will define our exposed population as individuals who signed the contract to receive a house from MCMV, and our non-exposed group will be comparable individuals within the cohort who have not signed a contract for a house at that time. We will estimate the effect of MCMV on health outcomes using different propensity score (PS) approaches to control for observed confounders. Follow-up time of individuals will begin at the date of exposure ascertainment and will end at the time a specific outcome occurs, date of death or end of follow-up (31st December 2015). In addition, we will conduct stratified analyses by follow-up time, age group, race/ethnicity, gender and socioeconomic position.

Ethics and dissemination

The study was approved by the ethic committees from *Instituto Gonçalo Muniz* – Oswaldo Cruz Foundation and University of Glasgow Medical, Veterinary & Life Sciences College. Data analysis will be carried out using an anonymised dataset, accessed by researchers in a secure computational environment according to the Center for Integration of Data and Health Knowledge (CIDACS) procedures. Study findings will be published in high quality peer-reviewed research journals and will also be disseminated to policy makers through stakeholder events and policy briefs.

Articles Summary

Strengths and limitations of this study

1. This *will be the first study to evaluate the effect of a major social housing programme on health outcomes in a middle-income country and is likely to be the largest of its type across the world*. This would allows to assess impacts on population subgroups, including adoption of an intersectionality perspective.

2. A comprehensive assessment of health impacts is being conducted, including both infectious and Non-Communicable Diseases (NCDs).

3. Our analytical approach includes the use of Propensity Score Matching, which has the limitation of accounting for only observed variables.

4. Health and behavioural information (such as smoking status, diet and comorbidities) prior to the intervention are not available, and there is therefore a risk of residual confounding arising from inadequate comparability between exposure groups.

5. Finally, this study does not allow estimating long-term effects of MCMV on health, *especially for NCDs, such as CVD mortality, and neglected diseases, such as leprosy,* given the limited length of follow-up available (up to eight years).

Introduction

Housing is a basic human right and an important social determinant of health and well-being, which not only includes the guarantee of shelter, but also its quality [1,2]. Several studies have investigated the relationship between population health and housing conditions, most of them in high-income countries [1,3–6]. Residential instability, crowding, temperature, and substandard housing conditions (such as water leaks, poor ventilation, dirty carpets, and pest infestation) are associated with chronic and infectious diseases [1,2,7,8]. The neighbourhood in which a house is located has also been shown to be associated with health in studies in high-income countries [9]. Physical neighbourhood characteristics that have been associated with health outcomes include: green parks, schools, health services, sidewalks, public transport, sanitation, aesthetic characteristic and connectivity of the street, bike lanes, availability and relative cost of healthy foods and tobacco [5,9–11]. Less visible but also important are social neighbourhood characteristics, which include measures of social network and support, violence, and social capital, especially in vulnerable communities in high-income countries [3,4,12].

Taking into account these relationships, there is a policy expectation that housing interventions could contribute to improve health and reduce social inequalities, especially among the most vulnerable [1,5,13]. Despite this, we are aware of little or no robust evidence on the positive and negative effects of housing conditions on cardiovascular disease (CVD) *and infectious diseases, such as* leprosy and tuberculosis. Understanding housing impacts on health in low and middle-income countries also remains particularly poorly understood.

In Brazil, there is a double burden of infectious and chronic diseases among the poorest people and, therefore, we will evaluate the effect of Minha Casa Minha Vida (MCMV), a social housing programme, both on mortality from CVD, the leading cause of death in the country, and on infectious diseases associated with poverty, like tuberculosis and leprosy [14,15]. In Brazil, 27% of all-cause of death is attributed to CVD, with most assigned to ischaemic heart disease and stroke [16,17]. Leprosy and tuberculosis are two of the most important infectious diseases in Brazil and affect predominantly vulnerable and marginalised populations [18–20]. Brazil has the second highest leprosy incidence worldwide, with almost 30,000 cases annually [14]. Tuberculosis is also common; the country reported 72,788 new cases in 2018 and 4,534 deaths in 2017 [14].

Given the social patterning of housing access and quality in Brazilian context, housing interventions may also have important impacts on the health of disadvantaged population groups, with potential benefits for racial/ethnic minorities, women, and individuals and families of lower socioeconomic position. We therefore aim to evaluate the health effects of the Brazilian social housing program MCMV, the largest social housing program in Latin American.

Our detailed objectives are:

1. To estimate the effect of MCMV on premature cardiovascular disease, ischaemic heart disease and, cerebrovascular disease mortality;

2. To estimate the effect of MCMV on all-cause mortality;

3. To estimate the effect of MCMV on leprosy and tuberculosis incidence;

4. To investigate whether any observed effects of MCMV on cardiovascular and all-cause mortality, leprosy and tuberculosis incidence differ by population subgroups (gender, race/ethnicity, age, socioeconomic position and length of follow-up).

Methods and analysis

Study design and population

This is a dynamic, retrospective, and open cohort study that will be drawn from individuals registered in the baseline of The 100 Million Brazilian Cohort [21,22], a cohort of individuals applying for government social programmes in Brazil.

Patient and public involvement

This research was done without public involvement. Public were not invited to comment on the study design and were not consulted to develop public relevant outcomes or interpret the results, since we use an administrative and deidentified dataset *and do not have permission to contact individuals. Study findings will be discussed with managers and specialists from the National Housing Secretariat from the Ministry of Regional Development and the published results will be disseminated to the public through the mass media. This study is a joint effort with the National Housing Secretariat from the Ministry of Regional Development, in order to guarantee that the findings would answer relevant policy questions. CIDACS staff are, in synergy with these key stakeholders and decision-makers, providing the methodological rigor needed to assure sound results. Findings will be incorporated into the National Housing Plan which is currently under development in Brazil. The National Housing*

Secretariat from the Ministry of Regional Development will not interfere in the analysis and results from studies planned in this protocol.

Intervention

We report intervention characteristics as suggested by the TIDieR-PHP template [23]. MCMV was implemented in July 2009 by the Brazilian Federal Government [24]. Its main goals are to reduce the housing shortage in Brazil (which exceeded six million houses in 2016), with 89% of unmet need concentrated among low-income families (defined as earning less than three times the minimum wage), and improve the construction sector through job generation and wider Brazilian economic growth [24]. By 2018, the government had contracted 5,164,075 and delivered 3,787,200 million house units, resulting in over six million people receiving housing across Brazil [25].

MCMV was structured to reach families from different income classes using three distinct eligibility criteria and subsidies. In this study, we focus on Class 1 subsidies, which targets low-income families, defined as households with less than three times the minimum wage (622,0 BRL in 2010, equivalent to USD 116,25) per month. Class 1 subsidies are divided in two subprogrammes targeting either urban or rural areas. In municipalities with more than 50 thousand inhabitants, the MCMV programme uses the Residential Lease Fund (FAR-MCMV) to build or acquire new housing units. Individuals eligible for Class 1 MCMV living in municipalities with less than 50,000 inhabitants or living in rural areas receive other forms of MCMV adapted to their context. For this study, we focus on FAR-MCMV, which is the largest subprogramme of MCMV. From 2009 to 2015, we estimate that FAR-MCMV delivered over 1.2 million house units across the country [25].

Eligibility criteria for FAR-MCMV include: *i)* the applicant must have a household income less than or equal to three times the minimum wage (without considering other social benefits, such as income from a conditional cash transfer program); *ii)* not be an owner, assignee or promising buyer of a residential property; and *iii)* not have received any previous housing benefits or grants for the purchase of construction materials [24]. Priority criteria for FAR-MCMV include: *i)* living in a hazardous or unhealthy area or being homeless; *ii)* belonging to a family headed by a lone mother (i.e. no male partner); *iii)* having a disabled person(s) in the household, with legal proof; and *iv)* having elderly people, aged 60 years old or over within the household [24].

Logic models

We created a logic model for each of the health outcomes studied in this protocol, informed by the existing literature, to describe the hypothesised mechanisms through which the MCMV may affect (a) CVD mortality (Figure 1), (b) leprosy new case detection (here and after, named as "incidence") (Figure 2), and (c) tuberculosis incidence (Figure 3). We identified, for each of the outcomes, pathways that are likely to operate through direct (physical house conditions) and indirect forms (housing neighbourhood effects and subjective aspects associated with house ownership).

a) Logic model for CVD mortality

The Programme may affect cardiovascular health through different pathways. Possible direct effects include changes in the physical standards of housing, leading to improvements in thermal and acoustic comfort, basic sanitation and household density reduction [2,26–28]. Indirect effects are related to the inclusion of families in new social and physical neighbourhood environments – due to the relocation process – with potentially better socio-economic, structural and physical contexts, as well as better access to basic health services [10,29,30]. On the other hand, relocation may negatively influence the social environment of the neighbourhood, since beneficiaries lose social networks and support, reducing social cohesion [3,12,31] (Figure 1).

In the short term, improvements in living conditions could enable changes in health behaviours, like physical activity, diet, alcohol and tobacco consumption [4,11]. In addition, reducing the cost of housing also provides greater access to resources which can be spent on healthier food and health care, leading to better control of cardiometabolic risk factors [32,33]. *In contrast, it is possible that greater availability of income could lead to greater consumption of unhealthy products (such as ultra-processed food, tobacco and alcohol) which could in turn increase cardiovascular risk* [34,35] (Figure 1).

In the long-term, the Programme could contribute to reducing chronic and cumulative exposure to psychosocial risk factors arising from inadequate housing contexts and this could potentially reduce the incidence of cardiovascular events [36,37]. The effects of the Programme on cardiovascular outcomes might be differential by age, race/ethnicity, socioeconomic position and gender, and might contribute to the reduction of disparities in cardiovascular mortality in Brazil (Figure 1).

Figure 1: Logic model to evaluate the effect of Minha Casa Minha Vida on reduction of cardiovascular mortality.

b) Logic model for leprosy

There is a strong relationship between housing and differential leprosy exposure [38–41]. Housing with better infrastructure and access to drinking water and adequate sanitation may improve hygiene conditions [18,42]. Reduction in household crowding leads to lower contacts among members of the family and, consequently, reduction of leprosy transmission [43,44] (Figure 2).

Access to neighbourhood amenities might improve access to health services, as well as improve community cohesion, which are important influences on leprosy risk [38,45]. Places to purchase healthy food can facilitate food security, which might result in improvements in the nutritional status of individuals and accelerate the immune response in leprosy infection [38,46] (Figure 2).

Housing ownership may give feelings of security and/or prestige. Also, it can provide greater availability of income for expenditure on potentially health-enhancing products such as food and health care [13,47–49]. It is known that access to health care is important to support strategies for self-care, case detection, timely diagnosis and treatment, and prevention of more severe forms of the disease [50,51] (Figure 2).

Figure 2: Logic model to evaluate the effect of Minha Casa Minha Vida on Leprosy Incidence.

c) Logic model for tuberculosis

The Programme may affect tuberculosis incidence through different pathways. Better housing leads to better ventilation and overall housing conditions, such as exposure to sunlight and reduced crowding, which reduce the possibility of transmission through aerial dispersion of the bacillus [52,53]. Alternatively, we may observe beneficiaries experiencing better quality of life or socioeconomic status due to a better environment [53]. Better housing location, if accompanied by more access to public services, including health care, can alter individual susceptibility to disease and improve treatment outcomes for those already infected. On the other hand, longer distances to school and workplace may lead to physical

stress, worsened immunity and predispose people to infection given exposure to *Mycobacterium tuberculosis* (MTB) [20,54]. Social support may also change due to the relocation, and may affect exposure to the agent as well as disease progression [55] (Figure 3).

We expect a differential effect of this housing programme by age group as the direct effects of the housing material will be more pronounced in children, especially in the case of intradomiciliary contacts of tuberculosis cases. As people grow older and become more exposed to external environment related factors, we hypothesise the effect of the programme to be the net effect of improved housing quality and surrounding area related factors (Figure 3).

Figure 3: Logic model to evaluate the effect of Minha Casa Minha Vida on tuberculosis incidence.

Datasets

We will link deterministically the baseline of the 100 Million Brazilian Cohort (2001-2015) [56], which includes information on socioeconomic and demographic variables, to the FAR-MCMV (2009-2015), the Mortality Information System (SIM) (2007-2015), and the leprosy (2007-2015) and tuberculosis (2007-2015) registries from the Notifiable Disease Information System [22,57,58]. The final deidentified dataset will contain information from recipients and non-recipients of the FAR-MCMV.

Sociodemographic variables

The 100 Million Brazilian Cohort includes baseline socioeconomic and demographic data from over 114 million individuals (approximately 55% of the Brazilian population in 2019). The cohort comprises people who are enrolled in the *Cadastro Unico*, a register containing all individuals within a household that have applied for any social programme administered by the Brazilian Federal Government [22]. We extracted individual level information (age, sex, race/ethnicity, education and occupation), household characteristics (household density, region and area of residence, household construction material, water supply, sanitation, electricity and waste disposal); and monthly per capita income for all family members [22].

Minha Casa Minha Vida Programme – FAR modality

Socioeconomic and demographic information (date of birth, gender, marital status, household monthly per capita income, name of head of household) from those who sign the contract to receive the housing unit – the main beneficiary – is obtained from the FAR-MCMV database [22]. In addition, we will extract from this database the name and Social identification number (SIN) from those who sign the contract, the date of signature of the contract (i.e., a *proxy* for the time that housing unit was delivered to the family), individuals who live with the main beneficiary at that time, and the address of the housing unit or the condominium delivered [22].

Mortality Information System (SIM)

Deaths within Brazil are subject to certification by medical professionals, so the causes of death (using ICD-10 codes) can be ascertained reasonably precisely. Despite the death registry being compulsory, there is evidence of under-ascertainment of deaths, particularly within areas that are more rural and in poorest regions of Brazil [59]. Coding of CVD related causes is thought to be good, but for some analyses, corrections are needed to take account of ill-defined causes [60]. In addition, since 2010 the proportion of garbage codes, causes of deaths that should not be considered as underlying causes of deaths, had declined, and in 2015, an estimation, show that 97.2% of deaths were included in the mortality system [60]. The highest ascertainment was observed in the state of São Paulo (99.8%), located in the Southeast region, and the lowest was observed in the state of Amapá (91.2%), in the North region of the country [60]. Under-ascertainment and miscoding of deaths is known to be most problematic in older adults, as well in young children [60].

Notifiable Diseases Information System (SINAN)

Leprosy and tuberculosis notification to the SINAN disease registry is compulsory in Brazil and coded using ICD-10. The records include individuals' socio-demographic and clinical information at the time of diagnosis and treatment updates when available. SINAN has improved its quality and completeness over time [61]. However, underreported cases and missing information still happen, especially in the poorest regions of the country. Therefore, leprosy and tuberculosis reporting to the SINAN notification system is based on passive surveillance and there is therefore heterogeneity in the frequency and completeness of reporting, which may result in the true incidence of diseases being underestimated [61].

Data analysis plan

Definitions of exposure and outcomes

We will define our exposed population as individuals who signed the contract to receive the housing unit from FAR-MCMV and the household members who live with them at the time of the contract signature. If there are no household members registered at the time of contract signature, we will include household members that appear in the last update of *Cadastro Único* carried out up to 2 years before receipt of the house unit from FAR-MCMV.

All outcomes will be defined according to the International Classification of Diseases, version 10 (ICD-10). We will evaluate the following outcomes: *i)* all-cause mortality; *ii)* CVD mortality (I00-I99); *iii)* ischaemic heart disease mortality (I20-I25); iv) cerebrovascular disease mortality (I60-I69); *v)* incidence of leprosy (A30); and *vi)* incidence of tuberculosis (A15-A19). All-cause and CVD mortality will be evaluated in adults aged 30 to 69 years old (defined as premature mortality within Brazil and of particular relevance to health policy) and in adults aged 18 to 69 years old (the broader adult population with the most reliable mortality data). We will not investigate outcomes among older adults, given the known issues of ascertaining mortality for this age group within the mortality information system. The incidence of leprosy and tuberculosis will be evaluated in all age groups.

To evaluate all-cause and specific cardiovascular mortality in individuals aged 18-69 years old, the follow-up time (in years) for each individual will start at entry into the analytical cohort (i.e. on signing a contract for the intervention group or the matching date for the control group) or the age at which an individual reaches 18 years of age, whichever is later. The follow-up time will end at the first of: date of death, end of follow-up (31st December 2015) or reaching 70 years of age.

To evaluate the incidence of leprosy and tuberculosis among individuals for all ages, the follow-up time (in years) for each individual will start at entry into the analytical cohort and will end at the first diagnosis of leprosy or tuberculosis, date of death or end of follow-up (31st December 2015).

Analysis

Estimating the effect of the FAR-MCMV programme on health outcomes is challenging due to selective uptake of the intervention by individuals. To address this, we will use different propensity score (PS) approaches to identify comparable individuals (based on observable characteristics) who did and did not receive the FAR-MCMV intervention, given individual-level characteristics [62]. Matching methods will include nearest neighbour matching using narrow callipers to minimise bias and Kernel matching [63,64]. In addition to matching methods, we will also estimate the effect of MCMV on the selected outcome using survival models weighted by the Inverse Probability of Treatment (IPTW) to estimate the average treatment effects (ATEs) and average treatment effect on the treated (ATTs) [62].

In addition, we will stratify the analyses for key subgroups of interest – namely, gender, race/ethnicity, socioeconomic position and phases of implementation of MCMV programme. We will also investigate whether effects differ across combinations of these characteristics. Specifically for leprosy, we will also stratify the analyses by residence in a high leprosy burden municipality as defined by the Brazilian Ministry of Health [65]. We will also stratify our analyses for individuals that are (and are not) beneficiaries of the *Bolsa Familia Programme* (BFP), one of the largest conditional cash transfer of the world [66].

Robustness checks

To check the robustness of our findings we will perform different tests. First, in the propensity score matching analysis, we will restrict our data from matching individuals with more narrow PS (different callipers). We will also restrict our analysis to certain types of municipality where data from MCMV-FAR or the mortality information system have better quality.

Sensitivity analyses

The matching strategy relies on the outcome being independent of treatment, conditional on the propensity score (Conditional Independence Assumption – CIA) [67]. However, if there are unobserved variables which affect assignment into treatment and the outcome variable simultaneously, a bias might arise. Since this assumption is non-testable by its nature, questions about the plausibility of the CIA can arise, and our results or at least their statistical significance could probably be driven by an omitted variable strongly correlated with the treatment outcome.

We will carry out sensitivity analyses to assess how strong the influence of these postulated unobservables might be in our study. We will use Rosenbaum bounds approach [68] and the sensitivity strategy proposed by Ichino et al [67]. This approach aims to assess the bias of our estimates when the CIA is assumed to fail in some meaningful way. A failure in the CIA is equivalent to saying that the assignment to treatment is not unconfounded given the set of observable variables [67].

Ethics and dissemination

The 100 Million Brazilian Cohort study was approved by the ethics committee of *Instituto Gonçalo Muniz – Oswaldo Cruz Foundation* (project number: 1.612.302) and the specific aims of this project was submitted for ethical approval in the same ethics committee. In addition, the University of Glasgow Medical, Veterinary & Life Sciences College Ethics Committee also approved the study (project number: 200190001). All data are linked in a safe room with access restricted to specified people only. After the data are linked and the linkage accuracy is calculated, researchers will have full access to the deidentified dataset. The dataset will be accessed by researchers upon application to a data curation committee with a detailed analysis plan. The dataset will receive a Digital Object Identifier (DOI), and full specification of how the dataset was created will be available online. All manuscripts will be published in high quality peer-reviewed open access journals and will also be disseminated to policy makers through stakeholder events and policy briefs.

Authors' contributions: SVK, AJFF, JMP wrote the first draft of the protocol. RR, RFO, CSST, MNS, RLF, MYT, RO, EMLA, SA, PC, LS, AHL and MLB contributed with additional material. All authors reviewed the final version.

Funding statement: This research was funded by the National Institute for Health Research (NIHR) (GHRG /16/137/99) using UK aid from the UK Government to support global health research. The Social and Public Health Sciences Unit is core funded by the Medical Research Council (MC_UU_12017/13) and the Scottish Government Chief Scientist Office (SPHSU13). SVK is funded by a NHS Research Scotland Senior Clinical Fellowship (SCAF/15/02). Cidacs/Fiocruz is supported by grants from CNPq/MS/Gates Foundation (401739/2015-5), the Wellcome Trust, UK (202912/Z/16/Z). The views expressed in this publication are those of the authors and not necessarily those of the NIHR, the UK Department of Health, CNPq/MS/Gates Foundation and the Wellcome Trust.

Competing interest's statement: The authors declare no competing interests.

Data availability statement: All data relevant to the study are included in the article or uploaded as supplementary information.

References

1 World Health Organization. *WHO housing and health guidelines.* 2018.

2 Braubach M, Jacobs DE, Ormandy D. Environmental burden of disease associated with inadequate housing. 2011.

3 Barber S, Hickson DA, Wang X, *et al.* Neighborhood disadvantage, poor social conditions, and cardiovascular disease incidence among African American adults in the Jackson heart study. *Am J Public Health* 2016;**106**:2219–26. doi:10.2105/AJPH.2016.303471

4 Wang X, Auchincloss AH, Barber S, *et al.* Neighborhood social environment as risk factors to health behavior among African Americans: The Jackson Heart Study. *Heal Place* 2017;**45**:199–207. doi:10.1016/j.healthplace.2017.04.002

5 Thomson H, Thomas S. Developing empirically supported theories of change for housing investment and health. *Soc Sci Med* 2015;**124**:205–14. doi:10.1016/j.socscimed.2014.11.043

6 Thomson H, Thomas S, Sellstrom E, *et al.* The health impacts of housing improvement: a systematic review of intervention studies from 1887 to 2007. *Am J Public Health* 2009;**99 Suppl 3**. doi:10.2105/ajph.2008.143909

7 Krieger J, Higgins DL. Housing and health: time again for public health action. *Am J Public Health* 2002;**92**:758–68.http://www.ncbi.nlm.nih.gov/pubmed/11988443%0Ahttp://www.pubmedcentral.nih.gov/articlerender.fcgi?artid=PMC1447157

8 Evans GW, Wells NM, Moch A. Housing and Mental Health: A Review of the Evidence and a Methodological and Conceptual Critique LK - https://tue.on.worldcat.org/oclc/437737953. *J Soc Issues TA - TT -* 2003;**59**:475–500.

9 Diez Roux A V. Neighborhoods and Health: What Do We Know? What Should We Do? *Am J Public Health* 2016;**106**:430–1. doi:10.2105/AJPH.2016.303064

10 Malambo P, Kengne AP, De Villiers A, *et al.* Built Environment, Selected Risk Factors and Major Cardiovascular Disease Outcomes: A Systematic Review. *PLoS One* 2016;**11**:e0166846. doi:10.1371/journal.pone.0166846

11 Poelman M, Strak M, Schmitz O, *et al.* Relations between the residential fast-food environment and the individual risk of cardiovascular diseases in The Netherlands: A nationwide follow-up study. *Eur J Prev Cardiol* 2018;**25**:1397–405. doi:10.1177/2047487318769458

12 Robinette JW, Charles ST, Gruenewald TL. Neighborhood cohesion, neighborhood disorder, and cardiometabolic risk. *Soc Sci Med* 2018;**198**:70–6. doi:10.1016/j.socscimed.2017.12.025

13 Bray N, Burns P, Jones A, *et al.* Costs and outcomes of improving population health through better social housing: a cohort study and economic analysis. *Int J Public Health* 2017;**62**:1039–50. doi:10.1007/s00038-017-0989-y

14 Brasil. Ministério da Saúde. Sala de Apoio à Gestão Estratégica do Ministério da Saúde (SAGE). Indicadores de morbidade: Hanseníase. Ministério da Saúde. 2019.http://sage.saude.gov.br/#

15 Brasil. Sala de apoio a gestão estratégica. Situação de saúde. Indicadores de morbidade: Tuberculose. Ministério da Saúde. 2019.https://sage.saude.gov.br/ (accessed 25 May 2020).

16 WHO. World Health Organization. Cardiovascular disease: prevention and control. 2009.http://www.who.int/dietphysicalactivity/publications/facts/cvd/en/

17 Brasil. Saúde Brasil 2018. 2018. http://bvsms.saude.gov.br/bvs/publicacoes/saude_brasil_2018_analise_situacao_saude_doencas_agravos_cronicos_desafios_perspectivas.pdf (accessed 15 Oct 2019).

18 Pescarini JM, Strina A, Nery JS, *et al.* Socioeconomic risk markers of leprosy in high-burden countries: A systematic review and meta-analysis. *PLoS Negl Trop Dis* 2018;**12**:1–20. doi:10.1371/journal.pntd.0006622

19 Dawson P, Perri BR, Ahuja SD. High Tuberculosis Strain Diversity Among New York City Public Housing Residents. *Am J Public Health* 2016;**106**:563–8. doi:10.2105/AJPH.2015.302910

20 Floyd K, Glaziou P, Zumla A, *et al.* The global tuberculosis epidemic and progress in care, prevention, and research: an overview in year 3 of the End TB era. Lancet Respir. Med. 2018;**6**:299–314. doi:10.1016/S2213-2600(18)30057-2

21 Centro de Integração de Dados e Conhecimento para a Saúde. The 100 Million Brazilian Cohort. 2019.https://cidacs.bahia.fiocruz.br/

22 Barreto ML, Almeida BDA, Ichihara MY, *et al.* The Center for Data and Knowledge Integration for Health (CIDACS). *Int J Popul Data Sci* 2019;**4**:1–24. doi:10.23889/ijpds.v4i2.1140

23 Campbell M, Katikireddi SV, Hoffmann T, *et al.* TIDieR-PHP: A reporting guideline for population health and policy interventions. *BMJ* 2018;**361**:1–5. doi:10.1136/bmj.k1079

24 Brasil. Lei Nº 11.977, de 7 de julho de 2009. Regulamenta o Programa Minha Casa Minha Vida. Brasil.: : http://www.planalto.gov.br/ccivil_03/_Ato2007-2010/2009/Lei/L11977.htm 2009. http://www.planalto.gov.br/ccivil_03/_Ato2007-2010/2009/Lei/L11977.htm (accessed 1 Sep 2019).

25 Brasil. Boletim mensal sobre os subsidios da União: Programa Minha Casa Minha Vida (10ª Edição). Brasília, Distrito Federal: 2019.

26 Gemmell I. Indoor heating, house conditions, and health. *J Epidemiol Community Heal* 2001;**55**:928–9. doi:10.1136/jech.55.12.928

27 Smith KR. Indoor air pollution in developing countries: recommendations for research. *Indoor Ari* 2003;**12**:198–207.

28 Sørensen M, Andersen ZJ, Nordsborg RB, *et al.* Road traffic noise and incident

myocardial infarction: A prospective cohort study. *PLoS One* 2012;**7**:1–7. doi:10.1371/journal.pone.0039283

29 Diez Roux A V, Borrell LN, Haan M, *et al.* Neighbourhood environments and mortality in an elderly cohort: Results from the cardiovascular health study. *J Epidemiol Community Health* 2004;**58**:917–23. doi:10.1136/jech.2003.019596

30 Brown AF, Liang L-JJ, Vassar SD, *et al.* Neighborhood socioeconomic disadvantage and mortality after stroke. *Neurology* 2013;**80**:520–7. doi:10.1212/WNL.0b013e31828154ae

31 Chaix B, Lindstrom M, Rosvall M, *et al.* Neighbourhood social interactions and risk of acute myocardial infarction. *J Epidemiol Community Heal* 2008;**62**:62–8. doi:10.1136/jech.2006.056960

32 Chambers EC, Hanna DB, Hua S, *et al.* Relationship between area mortgage foreclosures, homeownership, and cardiovascular disease risk factors: The Hispanic Community Health Study/Study of Latinos. *BMC Public Health* 2019;**19**:1–8. doi:10.1186/s12889-019-6412-2

33 Chambers EC, Rosenbaum E. Cardiovascular health outcomes of latinos in the affordable housing as an obesity mediating environment (AHOME) study: A study of rental assistance use. *J Urban Heal* 2014;**91**:489–98. doi:10.1007/s11524-013-9840-9

34 Martins APB, Monteiro CA. Impact of the Bolsa Família program on food availability of low-income Brazilian families: A quasi experimental study. *BMC Public Health* 2016;**16**:1–11. doi:10.1186/s12889-016-3486-y

35 Sperandio N, Rodrigues CT, Franceschini S do CC, *et al.* Impact of Bolsa Família Program on the nutritional status of children and adolescents from two Brazilian regions. *Rev Nutr* 2017;**30**:477–87. doi:10.1590/1678-98652017000400007

36 Robinette JW, Charles ST, Almeida DM, *et al.* Neighborhood features and physiological risk: An examination of allostatic load. *Heal Place* 2016;**41**:110–8. doi:10.1016/j.healthplace.2016.08.003

37 Erqou S, Echouffo-Tcheugui JB, Kip KE, *et al.* Association of cumulative social risk with mortality and adverse cardiovascular disease outcomes. *BMC Cardiovasc Disord* 2017;**17**:1–11. doi:10.1186/s12872-017-0539-9

38 Kerr-Pontes LRS, Barreto ML, Evangelista CMN, *et al.* Socioeconomic, environmental, and behavioural risk factors for leprosy in North-east Brazil: Results of a case-control study. *Int J Epidemiol* 2006;**35**:994–1000. doi:10.1093/ije/dyl072

39 Murto C, Chammartin F, Schwarz K, *et al.* Patterns of Migration and Risks Associated with Leprosy among Migrants in Maranhão, Brazil. *PLoS Negl Trop Dis* 2013;**7**. doi:10.1371/journal.pntd.0002422

40 Kumar A, Girdhar A, Yadav VS, *et al.* Some Epidemiological Observations on Leprosy in India. *Int J Lepr* 2001;**69**:234–40.

41 Nery JS, Ramond A, Pescarini JM, *et al.* Socioeconomic determinants of leprosy new case detection in the 100 Million Brazilian Cohort: a population-based linkage study. *Lancet Glob Heal* Published Online First: 2019. doi:10.1016/S2214-109X(19)30260-8

42 WHO/UNICEF. Progress on Sanitation and Drinking Water: 2015 Update and MDG

Assessment. 2015. doi:10.1007/s13398-014-0173-7.2

43 Fine PEM, Sterne JAC, Pönnighaus JM, *et al.* Household and dwelling contact as risk factors for leprosy in northern Malawi. *Am J Epidemiol* 1997;**146**:91–102. doi:10.1093/oxfordjournals.aje.a009195

44 Moet FJ, Pahan D, Schuring RP, *et al.* Physical Distance, Genetic Relationship, Age, and Leprosy Classification Are Independent Risk Factors for Leprosy in Contacts of Patients with Leprosy. *J Infect Dis* 2006;**193**:346–53. doi:10.1086/499278

45 Van Beers SM, De Wit MYL, Klatser PR. The epidemiology of mycobacterium leprae: Recent insight. *FEMS Microbiol Lett* 1996;**136**:221–30. doi:10.1016/0378-1097(95)00505-6

46 Teixeira CSS, de Medeiros DS, Alencar CH, *et al.* Nutritional aspects of people affected by leprosy, between 2001 and 2014, in semi-arid Brazilian municipalities. *Cienc e Saude Coletiva* 2019;**24**:2431–41. doi:10.1590/1413-81232018247.19642017

47 Howden-Chapman P, Chapman R. Health co-benefits from housing-related policies. *Curr Opin Environ Sustain* 2012;**4**:414–9. doi:10.1016/j.cosust.2012.08.010

48 Baker E, Mason K, Bentley R, *et al.* Exploring the Bi-directional Relationship between Health and Housing in Australia. *Urban Policy Res* 2014;**32**:71–84. doi:10.1080/08111146.2013.831759

49 Lindberg RA, Shenassa ED, Acevedo-Garcia D, *et al.* Housing interventions at the neighborhood level and health: a review of the evidence. *J Public Health Manag Pract* 2010;**16**:44–52. doi:10.1097/PHH.0b013e3181dfbb72

50 Barreto ML, Teixeira MG, Bastos FI, *et al.* Successes and failures in the control of infectious diseases in Brazil: Social and environmental context, policies, interventions, and research needs. Lancet. 2011;**377**:1877–89. doi:10.1016/S0140-6736(11)60202-X

51 Moschioni C, Antunes CM de F, Grossi MAF, *et al.* Fatores de risco para incapacidade física no momento do diagnóstico de 19.283 casos novos de hanseníase. *Rev Soc Bras Med Trop* 2010;**43**:19–22. doi:10.1590/S0037-86822010000100005

52 Wingfield T, Schumacher SG, Sandhu G, *et al.* The seasonality of tuberculosis, sunlight, vitamin D, and household crowding. *J Infect Dis* 2014;**210**:774–83. doi:10.1093/infdis/jiu121

53 Hargreaves JR, Boccia D, Evans CA, *et al.* The social determinants of tuberculosis: from evidence to action. *Am J Public Health* 2011;**101**:654–62. doi:10.2105/AJPH.2010.199505

54 Hayward SE, Dowd JB, Fletcher H, *et al.* A systematic review of the impact of psychosocial factors on immunity: Implications for enhancing BCG response against tuberculosis. *SSM - Popul Heal* 2020;**10**:100522. doi:10.1016/j.ssmph.2019.100522

55 Paz-Soldan VA, Alban RE, Jones CD, *et al.* The provision of and need for social support among adult and pediatric patients with tuberculosis in Lima, Peru: a qualitative study. *BMC Health Serv Res* 2013;**13**:290.

56 Barbosa GCG, Ali MS, Araujo B, *et al.* CIDACS-RL: a novel indexing search and scoring-based record linkage system for huge datasets with high accuracy and scalability. *BMC Med Inform Decis Mak* 2020;**20**:1–13. doi:10.1186/s12911-020-

01285-w

57 Sanni Ali M, Ichihara MY, Lopes LC, *et al.* Administrative data linkage in Brazil: Potentials for health technology assessment. *Front Pharmacol* 2019;**10**:1–20. doi:10.3389/fphar.2019.00984

58 Pita R, Pinto C, Sena S, *et al.* On the Accuracy and Scalability of Probabilistic Data Linkage over the Brazilian 114 Million Cohort. *IEEE J Biomed Heal Informatics* 2018;**22**:346–53. doi:10.1109/JBHI.2018.2796941

59 Franc¸a E, Teixeira R, Ishitani L, *et al.* Ill-defined causes of death in Brazil: A redistribution method based on the investigation of such causes. *Rev Saude Publica* 2014;**48**:671–81. doi:10.1590/S0034-8910.2014048005146

60 IBGE. Instituto Brasileiro de Geografia e Estatística. Informação Demográfica e Socioeconômica. Sistemas de estatísticas vitais no Brasil: avanços, perspectivas e desafios. 2018. https://biblioteca.ibge.gov.br/visualizacao/livros/liv101575.pdf

61 Brasil. Ministério da Saúde. Secretaria de Vigilância à Saúde. Guia de Vigilância Epidemiológica. 2005. http://portal.anvisa.gov.br/documents/33916/388729/Guia_Vig_Epid_novo2.pdf/99464018-d6d1-486b-853b-9871d6eff16f?version=1.0

62 Austin PC. The performance of different propensity score methods for estimating marginal hazard ratios. *Stat Med* 2013;**32**:2837–49. doi:10.1002/sim.5705

63 Lunt M. Selecting an appropriate caliper can be essential for achieving good balance with propensity score matching. *Am J Epidemiol* 2014;**179**:226–35. doi:10.1093/aje/kwt212

64 Baser O. Too much ado about propensity score models? Comparing methods of propensity score matching. *Value Heal* 2006;**9**:377–85. doi:10.1111/j.1524-4733.2006.00130.x

65 Brasil. Ministério da Saúde. Sistema de Legislação da Saúde. Portaria nº 2.556, de 28 de outubro de 2011. Avaiable from:

66 Soares FV, Ribas RP, Osório RG. Evaluating the impact of Brazil's Bolsa Família: Cash transfer programs in comparative perspective. *Lat Am Res Rev* 2010;**45**:173–90.http://www.undp-povertycentre.org

67 Ichino A, Mealli F, Nannicini T. From temporary helps jobs to permanent employment: what can we learn from matching estimators and their sensitivity? *J Appl Econom* 2008;**23**:305–27. doi:10.1002/jae

68 Rosenbaum P. *Observational Studies*. New York: : Springer US 2002.

Figure 1: Logic model to evaluate the effect of Minha Casa Minha Vida on reduction of cardiovascular mortality.

Figure 2: Logic model to evaluate the effect of Minha Casa Minha Vida on Leprosy Incidence.

Figure 3: Logic model to evaluate the effect of Minha Casa Minha Vida on tuberculosis incidence.

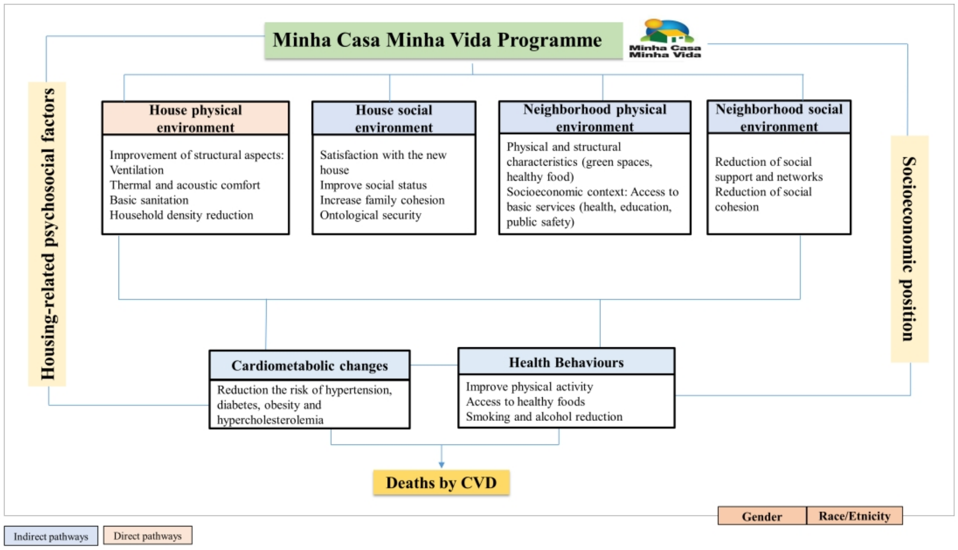

338x190mm (300 x 300 DPI)

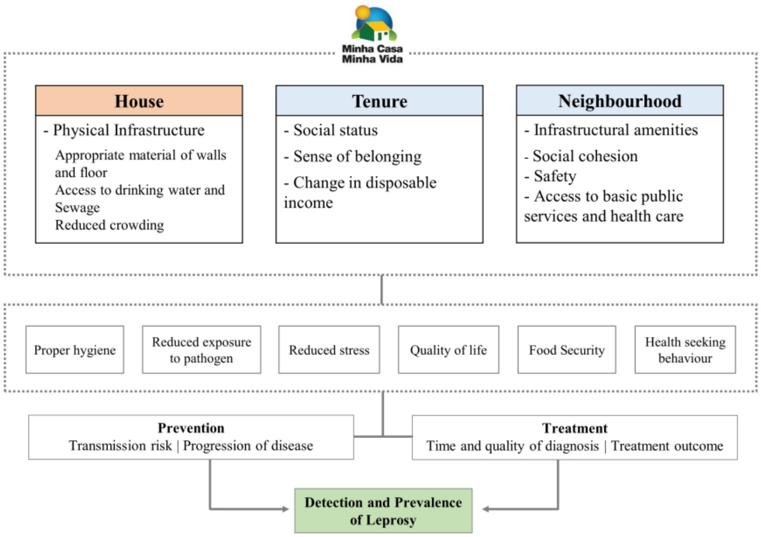

338x190mm (300 x 300 DPI)

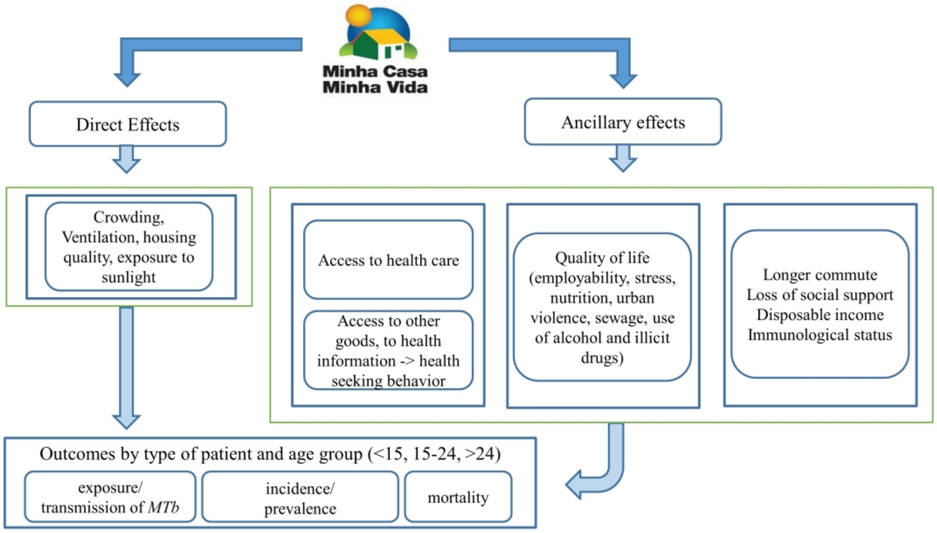

338x190mm (300 x 300 DPI)
